# Identification and odor exposure regulation of odorant-binding proteins in *Picromerus lewisi*

**DOI:** 10.3389/fphys.2024.1503440

**Published:** 2024-12-04

**Authors:** Shan-Cheng Yi, Jia-Ling Yu, Sara Taha Abdelkhalek, Zhi-Rong Sun, Man-Qun Wang

**Affiliations:** ^1^ Hubei Insect Resources Utilization and Sustainable Pest Management Key Laboratory, College of Plant Science and Technology, Huazhong Agricultural University, Wuhan, China; ^2^ Department of Entomology, Faculty of Science, Ain Shams University, Cairo, Egypt; ^3^ Southwest Guizhou Autonomous Prefecture Tobacco Company, Xingren, China

**Keywords:** *Picromerus lewisi*, *Spodoptera litura*, odor exposure, transcriptome, odorantbinding proteins

## Abstract

The highly developed sensitive olfactory system is essential for *Picromerus lewisi* Scott (Hemiptera: Pentatomidae) adults, an widely distributed natural predatory enemy, to locate host plants. During this process, odorant-binding proteins (OBPs) are thought to have significant involvement in the olfactory recognition. However, the roles of OBPs in the olfactory perception of *P. lewisi* are not frequently reported. Here, we conducted odor exposure and transcriptome sequencing experiments using healthy and *Spodoptera litura*-infested tobacco plants as odor sources. The transcriptomic data revealed that the alteration in the expression of mRNA levels upon exposure to odor was sex-dependent. As the expression profiles differed significantly between male and female adults of *P. lewisi*. A total of 15 *P. lewisi* OBPs (PlewOBPs) were identified from the *P. lewisi* transcriptome. Sequence and phylogenetic analysis indicated that PlewOBPs can be classified into two subfamilies (classic OBP and plus-C OBP). The qRT-PCR results showed that the transcript abundance of 8 *PlewOBPs* substantially altered following exposure to *S. litura*-infested tobacco plants, compared to the blank control or healthy plants. This implies that these *PlewOBPs* may have an olfactory function in detecting *S. litura*-infested tobacco plants. This study establishes the foundation for further understanding of the olfactory recognition mechanism of *P*. *lewisi* and helps discover novel targets for functional characterization in future research.

## 1 Introduction

Insects utilize a diverse array of molecular sensors to detect and respond to their external environment (Gadenne et al., 2016; Hare, 2011; Howe and Jander, 2008). In this process, various chemosensory-related proteins are involved in the transduction of signals within antennae, such as odorant-binding proteins (OBPs), chemosensory proteins (CSPs), odorant receptors (ORs), odorant degrading enzymes (ODEs), and sensory neuron membrane proteins (SNMPs) (Leal, 2013; Paoli and Galizia, 2021; Tunstall et al., 2012). The OBPs are a family of compact soluble proteins (12–30 kDa) that selectively bind and transport hydrophobic molecules across the hydrophilic sensillum lymph surrounding sensory neurons (Tunstall et al., 2012; Rihani et al., 2021; Pelosi et al., 2018; Sun et al., 2018). This ultimately leads to the activation of odorant recptors (ORs).

Insect OBPs are characterized by the presence of three interlocking disulfide bonds, which are formed by highly conserved cysteine (Cys) residues. OBPs can be categorized into four subfamilies based on the number of preserved Cys residues: classic OBPs (have six conserved Cys residues), minus-C OBPs (have four conserved Cys residues), plus-C OBPs (have eight conserved Cys and proline residues), and atypical OBPs (have 9–10 Cys residues and a long C-terminus) (Manoharan et al., 2013; Vieira and Rozas, 2011; Zhou et al., 2010; Vieira et al., 2007). Multiple investigations have confirmed that OBPscan selectively recognize and evaluate specific chemical signals (Yang et al., 2024; Xiang et al., 2023; Sims et al., 2023; Li E.-T. et al., 2023; Huang et al., 2023). Thus, their function goes beyond just passive transportation. The knockdown of *BdorOBP83a-2* in *Bactrocera dorsalis* (Diptera: Trypetidae) resulted in a significant reduction of 60%–70% in the electroantennogram (EAG) response to methyl eugenol (Wu et al., 2016). It also caused a 30%–50% increase in flight time to reach the odor source. This study demonstrates that BdorOBP83a-2 plays a crucial role in mediating the responses of the oriental fruit fly to semiochemicals (Wu et al., 2016). The OBP3 identified in *Acyrthosiphon pisum* (Hemiptera: Aphididae) has an important function in the discrimination of the alarm pheromone (*E*)-β-farnesene compared to its homologous proteins OBP1 and OBP8 (De Biasio et al., 2015). *Drosophila melanogaster* flies with *OBP57e* and *OBP57d* knock-out displayed altered behavioral responses to hexanoic and octanoic acids (Yasukawa et al., 2010; Matsuo et al., 2007; Harada et al., 2008). Furthermoe, when *OBP57d* and *OBP57e* from *D. simulans* and *D. sechellia* were introduced, the preference for the oviposition site in *D. melanogaster OBP57d/e* ^*KO*^ flies shifted to match that of the original species. These studies indicated that OBPs were essential for mediating olfactory behavioral responses.

The transcriptional expression of the majority of *OBPs* is mainly restricted to the antennae, maxillary palp, and proboscis of the insect’s head (Yi et al., 2023; Wang et al., 2020; Dong et al., 2023). Studies showed that exposing the olfactory system to external odorant cues alters the transcriptional levels of chemosensory-related proteins involved in detecting of the tested odorant (He et al., 2023; Anton and Rössler, 2021; Guerrieri et al., 2012). Discovery of this phenomenon originated from investigations on the deorphanization of ORs in *Mus musculus* (Rodentia: Muridae), employing a mechanism appropriately known as deorphanization of receptors based on expression alteration of mRNA levels (DREAM) (von der Weid et al., 2015). To date, this strategy has been effectively applied in several studies of insect OBPs. In *Diaphorina citri* (Hemiptera: Chermidae), *DcitOBP7* showed significant changes in expression levels, either upregulation or downregulation induced by methyl salicylate, linalool, and R-(+)-limonene. Moreover, the suppression of messenger RNA (mRNA) expression of *DcitOBP7* using the RNA interference (RNAi) resulted in a significant reduction in EAG activity and adult behavioral responses of *D. citri* to tested volatiles and the preferred host, *Murraya paniculata* (Sapindales: Rutaceae) (Liu et al., 2021). In *Holotrichia oblita* (Coleoptera: Melolonthidae), *HoblOBP13* and *HoblOBP9* were upregulated upon exposure to one of the female attractants (*E*)-2-hexenol and phenethyl alcohol. The female beetles that performed post-RNAi treatments targeting *HoblOBP13* and *HoblOBP9* exhibited an apparent reduction in attraction towards (*E*)-2-hexenol and phenethyl alcohol compared to water-injected beetles and those treated with GFP-dsRNA (Yin et al., 2019). Although the DREAM strategy sometimes showed a high amount of false positive predictions, it could still provide insights of the chemical communication between insects and their external environment (Koerte et al., 2018).

*Picromerus lewisi* (Hemiptera: Pentatomidae) is an important natural enemy insect with strong predation on the larvae of various lepidopteran pests, including *Spodoptera litura*, *Spodoptera frugiperda*, *Mythimna separate*, and *Leucania separata*. A previous study has identified the expression profiles of cytochrome P450 monooxygenases, carboxylesterase, and glutathione S-transferase genes across various tissues and developmental stages (Li et al., 2022; Li W. et al., 2023). Our previous investigations using a Y-tube olfactometer showed that *P. lewisi* adults had a significant preference for *S*. *litura*-infested tobacco plants compared to the healthy ones (unpublished data). However, the specific olfactory mechanisms underlying this behavioral preference remain unclear. In this study, we conducted transcriptome sequencing on the head of *P. lewisi* following exposure to healthy and *S*. *litura*-infested tobacco plants. A total of 18 *P*. *lewisi* libraries were constructed and sent for *de novo* assembly. Subsequently, we performed identification and phylogenetic analysis of *PlewOBPs*. Finally, we analyzed the differential expression profiles of different treatments on male and female *P*. *lewisi*, and validated the transcription abundance of all identified PlewOBPs using qRT-PCR. The provided results establish the foundation for further comprehension of the olfactory recognition mechanism of *P*. *lewisi*.

## 2 Materials and methods

### 2.1 Materials

The adult *P*. *lewisi* samples were reared in a controlled environment chamber with a temperature of 25°C ± 3°C, relative humidity (RH) of 65% ± 5%, and a light-dark cycle of 14:10 (L:D). The first-hatched *P*. *lewisi* larvae were fed with 10% honey water. After the 2^nd^ larval instar, *P. lewisi* larvae were provided with *L*. *separata* larvae of the corresponding instar. At the same time, *S*. *litura* larvae were fed on fresh tobacco leaves in an artificially controlled environment maintained at 25°C ± 3°C, 40% ± 5% RH, and natural light only.

The Yunnan Tobacco 87 plants were cultivated in a controlled environment with a temperature of 25°C ± 3°C, RH of 65% ± 5%, and a light-dark cycle of 14 L:10 D. The seeds were planted in seedling trays (a hole depth of 5 cm, an upper aperture of 4 cm, and a lower aperture of 2 cm) for 30 days. Subsequently, the seedlings were transferred to two-color pots (upper aperture of 14 cm, lower aperture of 12 cm, and height of 13 cm), and then the plants continued to grow for 30 days. Tobacco plants exhibiting robust and healthy growth and devoid of any pests and diseases were selected for the experiments.

### 2.2 Odor exposure and tissue collection

Following 6 h of starvation, six 4^th^ instar larvae of *S*. *litura* were affixed to the 3^rd^ and 4^th^ leaves of tobacco plants in a top-to-bottom manner. To prevent the escape of *S*. *litura* larvae, the plants were enclosed in cages with 120 mesh screens. The *S*. *litura* larvae were removed after consuming 20% of the leaves. In exposure experiments, tobacco plants were used 18–30 h after the *S. litura* commenced feeding. The root and soil portion of the plants were wrapped in tin foil and placed in a hermetically sealed glass container. The airflow from the air pump passed consecutively through two closed containers, one filled with activated carbon and the other withpure water. The purified and humidified air was directed into the glass containers housing tobacco plants and then out through the air outlet, with the flow rate being regulated by an airflow meter. Once the device was connected, the flow rate of the flow meter was adjusted to 400 mL/min, and the test proceeded once the flow rate had reached an equilibrium level. The gas maintained a consistent flow rate throughout the treatmentand was directly directed into the preservation box housing the *P. lewisi* adults.

A total of three distinct odor sources for conducting the odor exposure experiments. The first group served as a control, devoid of any odorants (CK group). The healthy and *S*. *litura*-infested tobacco plants were used as the source of odor in the HT and IT group, respectively. Each odor exposure experiments group comprises 15 mature *P. lewisi* individuals (male or female), with three biological replicates. After 1 h of odor exposure treatment, the entire head of the *P. lewisi* adult samples was collected and promptly placed in RNase-free tubes and stored in liquid nitrogen.

### 2.3 RNA extraction and cDNA synthesis

Total RNA was extracted from preserved tissues using TRIzol reagent (Invitrogen, Carlsbad, CA, United States) following the manufacturer’s instructions. The integrity and quantity of RNA samples were assessed by using 1% agarose gel electrophoresis and a spectrophotometer (Eppendorf Bio Photometer Plus, Hamburg, Germany), respectively. A complementary DNA (cDNA) was synthesized from 1 μg of total RNA via reverse transcription, using a Prime-Script II 1st Strand cDNA Synthesis Kit (TaKaRa Bio, Otsu, Japan) according to the pamphlet instructions. This kit uses DNase in the initial step to eliminate the effect of DNA on qRT-PCR.

### 2.4 Transcriptome analysis

All samples were conveyed to Personalbio Technology Co. Ltd. (Shanghai, China) for Illumina sequencing using a 100 bp paired-end sequencing strategy. The sequencing data was filtered to obtain high-quality, clean reads for subsequent analysis. For unreferenced transcriptome sequencing, the clean reads were transcribed using Trinity software for further analysis. The unigenes were functionally annotated using the NR, GO, KEGG, eggNOG, Swiss-Prot, and Pfam databases. The number of reads for each sample was compared to each gene to calculate the FPKM value. Differentially expressed genes were identified by the DESeq software package, with thresholds of log2 (fold change) ≥ 1, and *P*-value ≤0.05 set significant differences.

### 2.5 Identification and phylogenetic analysis of odorant-binding proteins

The transcriptome annotation results were used to select the unigenes that included the annotated content of “odorant-binding protein”, “odorant binding protein”, and “OBPs”. The identification of potential open reading frames (ORFs) and their corresponding amino acid sequences was determined using the ORF FINDER (https://www.ncbi.nlm.nih.gov/orffinder/). Hemipteran-based phylogenetic analysis of PlewOBPs was performed using the MEGA-X program. The N-terminal signal peptide sequences of PlewOBPs were predicted using the SignalP-5.0 server (http://www.cbs.dtu.dk/services/SignalP/). The mature amino acid sequences were aligned using the MAFFT multiple sequence alignment (https://mafft.cbrc.jp/alignment/server/). A neighbor-joining tree was constructed using the p-distance model and pairwise deletion of gaps. The bootstrapping process was performed by resampling the amino acid positions of 1,000 replicas, and branches with bootstrap cutoff of <50% were collapsed. The tree was ultimately examined and modified using the Evolview-v2 (https://evolgenius.info//evolview-v2/) and Adobe Illustrator CC 2019 program.

### 2.6 qRT-PCR

The relative mRNA expression levels of 15 full-length *PlewOBPs* genes were determined using qRT-PCR under various treatments. A qRT-PCR reaction was performed on a LightCycler^®^ 96 System using the Hieff^®^ qPCR SYBR Green Master Mix (No Rox) (Yeasen, Shanghai, China). Each reaction was systematically run in triplicates with three independent biological replicates. Gene-specific primers of *PlewOBPs* were designed using the Primer-BLAST service (https://www.ncbi.nlm.nih.gov/tools/primer-blast/index.cgi?LINK_LOC=BlastHome). The comparative 2^−ΔΔCT^ method was used to calculate the relative transcript levels in each sample. The data was assessed using a one-way analysis of variance (ANOVA) and Tukey’s honestly significant difference (HSD) *post hoc* test.

## 3 Results and discussion

### 3.1 Head transcriptome of *Picromerus lewisi*

**FIGURE 1:**
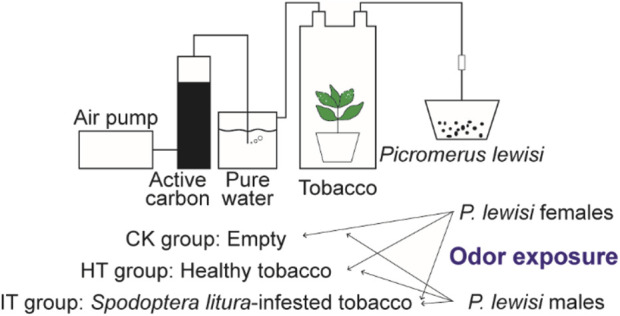
Schematic diagram of the odor exposure experiments.

A grand number of 765,095,950 Illumina paired-end reads were generated from 18 *P*. *lewisi* libraries. Each library was sequenced using samples obtained from the heads of 15 adult individuals (female or male) (Figure 1). After the trimming of adaptors and the filtration of low-quality reads, a total of 754,299,698 high-quality reads were obtained with 96.79% Q30 bases. Subsequently, we used the entire set of reads to create a *de novo* transcriptome assembly using Trinity (see methods). The assembly process resulted in the formation of 156,008 transcripts, which collectively had 276 million base pairs (bp). These transcripts encoded a total of 75,039 unigenes, with an N50 value of 1728 bp (Supplementary Table S1).

**FIGURE 2:**
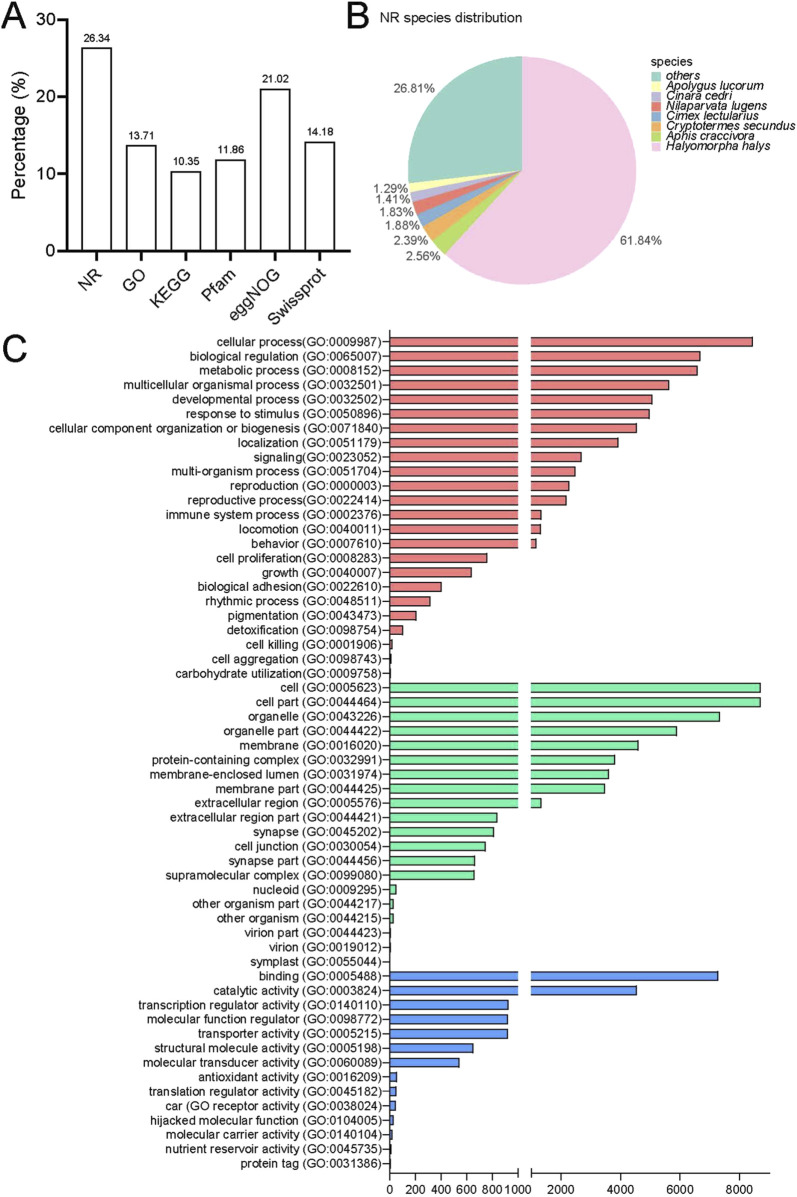
Annotation information for the head transcriptome of *Picromerus lewisi*. **(A)** Summary of annotations in different databases. **(B)** Species distribution in the NR database. **(C)** Functional classification of GO annotations.

The annotation process involved using BLAST and HMMER software to perform local alignments against various databases, including NR, GO, KEGG, Pfam, eggNOG, and Swissprot. This resulted in annotation for 19,762 (26.34%), 10,287 (13.71%), 7,766 (10.35%), 8,898 (11.86%), 15,770 (21.02%), and 10,639 (14.18%) unigenes, respectively (Figure 2A). Out of the total data set, 55,277 unigenes (75.37%) did not receive a BlastX hit, possibly due to misassembly or insufficient representation in the NR database. The assembled transcriptome of *P*. *lewisi* showed a significant resemblance to *Halyomorpha halys* (Hemiptera: Pentatomidae), with 12,221 (61.84%) of the annotated unigenes showing the closest similarity to that particular species (Figure 2B). It was expected as *H. halys* has the most thoroughly annotated genome among of stink bugs in the pentatomidae family (Sparks et al., 2020; Paula et al., 2016).

Further functional classification of gene ontology (GO) was performed. The GO database consists of three major categories: biological processes (BP), cellular components (CC) and molecular function (MF). In total, 24 biological processes, 20 cellular components and 14 molecular functions were identified (Figure 2C). The categories more directly related to insect olfaction in the list of BP were a response to stimulus and localization. The unigenes exhibited enrichment in cellular processes, biological regulation and metabolic processes in the BP. Most of the unigenes in the CC list were associated with cells, cell components, and organelles. Finally, in the list of MF, these were annotated to the functional classes of binding, catalytic activity, and transcription regulator activity. These predominant GO annotations in BP, CC, and MF were comparable to those observed in the antennal transcriptomes of the *H*. *halys*, *Adelphocoris lineolatus* (Hemiptera: Miridae), and *Apolygus lucorum* (Hemiptera: Miridae) (Paula et al., 2016; Xiao et al., 2017; Chen et al., 2017; Ji et al., 2013). This similarity is noteworthy, considering that the transcriptomes were obtained from the head of *P*. *lewisi*. However, the annotations differed from the predominant BP and MF detected in the antennal transcriptomes of *Chinavia ubica* (Hemiptera: Pentatomidae), *Dichelops melacanthus* (Hemiptera: Pentatomidae), or *Euschistus heros* (Hemiptera: Pentatomidae) (Farias et al., 2015). The GO enrichment variations could be attributed to their distinct dietary preferences.

### 3.2 Identification of odorant-binding proteins

OBPs are compact, globular, water-soluble proteins with a signal peptide at the N-terminal region and six Cys residues in conserved positions (Zhou et al., 2008). The Cys motif is a highly conserved tertiary protein structure consisting of six α-helices coordinated by three disulfide bridges (Zhou et al., 2004; Xu et al., 2003; Wang et al., 2014). It is commonly employed as a signature for the identification of OBP. Based on the sequence similarity to insect OBPs, a total of 15 PlewOBPs were identified in the transcriptome. The number of *PlewOBPs* is much lower compared to *H*. *halys* (30 HhalOBPs), *A*. *lineolatus* (31 AlineOBPs), and *A*. *lucorum* (38 AlucOBPs) (Paula et al., 2016; Yuan et al., 2015; Xiao et al., 2018; Gu et al., 2011). A plausible explanation is that their total RNA is more comprehensive than ours due to the extraction of RNA from various all developmental stages or tissues or because they possess whole genome sequences. *Picromerus lewisi* had a similar number of OBPs to other stink bugs, specifically 19 in *Tropidothorax elegans* (Hemiptera: Lygaeidae), 18 in *Cyrtorhinus lividipennis* (Hemiptera: Miridae), and 17 in *Rhodnius prolixus* (Hemiptera:Reduviidae) (Song et al., 2018; Wang et al., 2018; Wang et al., 2017; Oliveira et al., 2017).

All 15 *PlewOBPs* have intact ORFs with lengths ranging from 396 to 663 bp. The authenticity of the nucleotide sequences of all *PlewOBPs* was confirmed by cloning and sequencing. Out of the 15 PlewOBPs, 11 of them have a signal peptide at their N-terminal. Like other hemipterans OBPs, PlewOBPs sequences were categorized into two types based on the presence of the characteristic OBP Cys signature: “classic” OBP and “plus-C” OBP. Based on the hemipteran “classic” OBP Cys motif (C1-X_22-32_-C2-X_3_-C3-X_36-46_-C4-X8-14-C5-X_8_-C6), we have classified 12 PlewOBPs (PlewOBP 1–8, 10–12, and 15) sequences as “classic” OBPs (Supplementary Figure S1). The remaining three PlewOBP proteins (PlewOBP 9, 13, and 14) belong to the “plus-C” OBP family (Supplementary Figure S1), and fit to the Cys spacing pattern (C1-X_8-41_-C2-X_3_-C3-X_39-47_-C4-X_17-29_-C4a-X_9_-C5-X_8_-C_6_-P-X_9-11_-C6a).

**FIGURE 3:**
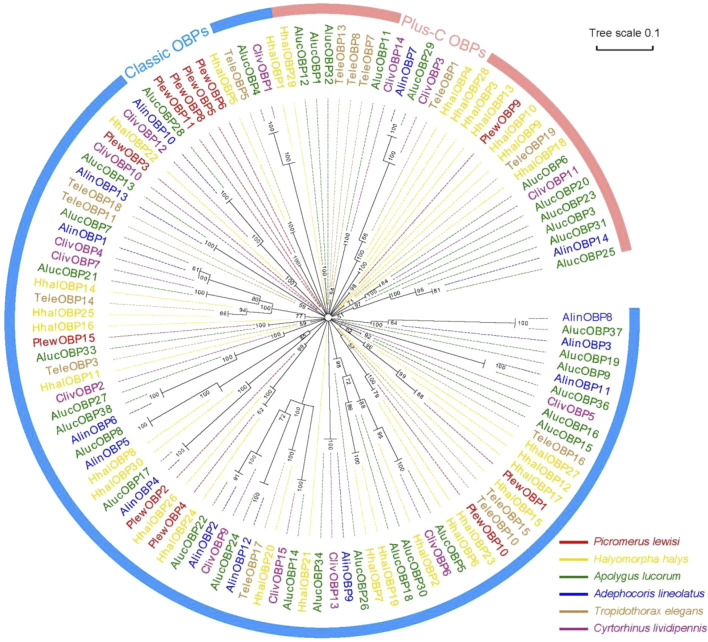
Phylogenetic analysis of odorant-binding proteins (OBPs) in hemipterans. Phylogenetic tree of OBPs from *Picromerus lewisi* and other hemipteran bugs: Plew, *P. lewisi*; Alin, *Adelphocoris lineolatus*; Aluc, *Apolygus lucorum*; Cliv, *Cyrtorhinus lividipennis*; Hhal, *Halyomorpha halys*; Tele, *Tropidothorax elegans*.

A neighbour-joining tree consisting of 119 mature OBPs was constructed from six Hemipteran species to confirm the intraspecific divergence of their OBPs. The Hemipteran OBP protein family generates an expansive tree, with distinct clades for both “classic” and “plus-C” OBP sequences (Figure 3). In the phylogenetic tree, OBPs of the same subfamily exhibit local clustering, while OBPs of the same subfamily are distributed evenly throughout the entire tree, forming separate central clusters. These results provide evidence of significant duplication and specialization of OBPs within Heteroptera. However, this finding diverges from aphids, which have the most orthologous sequences in different species (Xue et al., 2016; Wang et al., 2019; Song et al., 2021). In this work, we found no intraspecific orthologous genes within the same species of stink bugs. Nevertheless, there is a slightly higher rate of orthologues between related species. 10 of 15 PlewOBPs have homologous sequences to the OBPs found in other Hemiptera insects with a high bootstrap value, indicating a high probability that these sequences come from a common ancestor and are preserved for shared functions in plant bug species. PlewOBPs also have paralogs, such as PlewOBP5 and PlewOBP6, which may have undergone horizontal duplication duplicated from the same ancestor through natural selection to acquire an additional function.

### 3.3 Overall differential expression profiles

**FIGURE 4:**
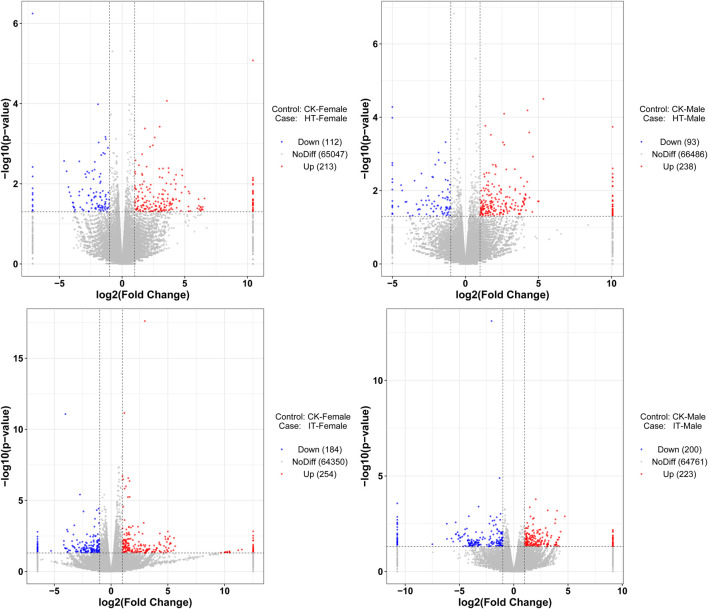
Volcano plot of RNA sequencing from different treatments. The vertical dashed lines represent the 2-fold expression difference thresholds; the horizontal dashed line represents the *p*-value = 0.05 threshold. Red dots indicate genes with significant upregulation, blue dots indicate genes with significant downregulation, and gray dots indicate genes with non-significant differential expression.

The identification of differentially expressed genes (DEGs) was carried out by the DESeq software package, with an absolute log2 (fold change) value of ≥1 and a *P*-value ≤0.05 were considered as the threshold for significant differences. Within this framework, the transcript abundance of 325 genes was significantly altered when females were exposed to volatiles emitted by healthy tobacco plants for 1 h. 112 genes demonstrated lower abundance, while 213 showed more abundance when compared to blank control (Figure 4A). Similarly, in males, a total of 93 genes showed decreased abundance out of the 331 genes that were differentially detected. Conversely, 238 genes revealed increased abundance in the HT group compared to the control group (Figure 4B). The *S*. *litura*-infested tobacco plants exhibited a higher propensity for genes to change in transcript abundance than healthy plants. Exposure of adult females to infested tobacco resulted in the upregulation of 254 genes and the downregulation of 184 ones (Figure 4C). Additionally, 223 genes were upregulated, and 200 were downregulated in adult males (Figure 4D).

**FIGURE 5:**
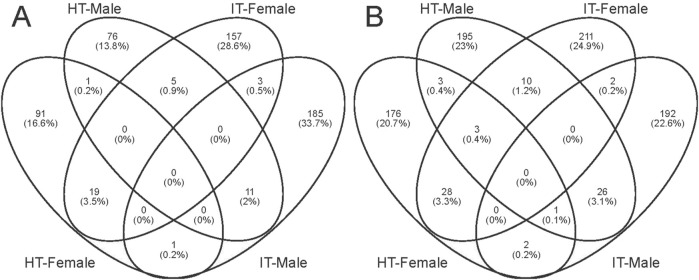
Wayne diagrams of differentially expressed genes. **(A, B)** Wayne diagrams of genes with significant downregulation and upregulation, respectively.

We conducted GO analysis to better understand the genes responsible for the variation between sexes and various treatments. Upon assessing the transcripts in male and female bugs, we observed distinct differences in the GO enrichment patterns of differentially expressed genes induced by healthy or infested tobacco plants (Supplementary Figures S2–S5). There was minimal overlap of GO enrichment terms between males and females within the same treatment and across different treatments within the same sex. The Wayne diagram analysis corroborated these findings, revealing that only a few genes were simultaneously up- or downregulated between different treatments or sexes (Figure 5). Among which, the venom serine protease-like gene was the only annotated gene, potentially serving common roles in digestion and detoxification (He et al., 2024). It was unsurprising that genes can respond to environmental factors, leading to alterations in transcript abundance *in vivo*. The transcript abundance of a large variety of genes in *Anopheles gambiae* (Diptera: Culicidae) mosquitoes can be influenced by varied light conditions (Rund et al., 2013). Whereas transcriptomic data in *Aedes aegypti* (Diptera: Culicidae) revealed that prolonged exposure to 1-octen-3-ol modulated ORs and OBPs genes, as well as cytochrome P450 enzymes, insect cuticle proteins, and glucuronosyltransferases families (Mappin et al., 2023). Abiotic environmental factors and biological factors such as courtship, mating, sex, and age can influence antennal chemosensory-related genes (Tallon et al., 2019; Alonso et al., 2019; Andersson et al., 2014; Siju et al., 2010). However, the transcriptome data in the present study revealed none differentially expressed *PlewOBPs*. One potential explanation is that this change in transcript levels correlates with the concentration and duration of odor exposure. The duration and concentration of our odor exposure experiments were insufficient (Duan et al., 2023). Conversely, minimal fold differences in gene expression may evade detection using transcriptome sequencing, as our screening criteria were based on thresholds of log_2_ (fold change) ≥ 1. To gain a more comprehensive understanding of the pattern of changes in the *OBP* gene family, we performed qRT-PCR experiments with all *PlewOBPs*.

### 3.4 Expression profile of PlewOBPs after exposure to tobacco

**FIGURE 6:**
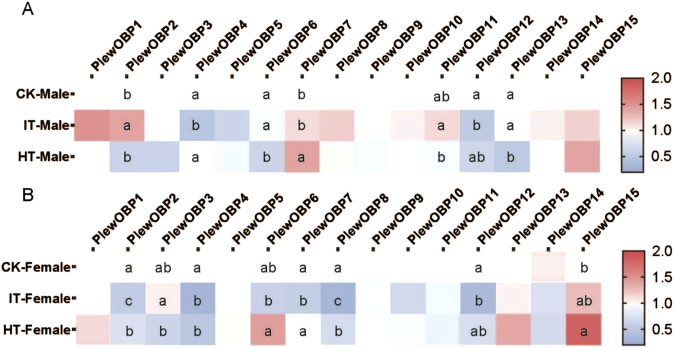
Heat map of expression profiles of PlewOBPs after odor exposure treatment. **(A, B)** The expression levels of PlewOBPs from male and female *Picromerus lewisi*, respectively. The results were analyzed using one-way ANOVA with Tukey’s HSD *post hoc* test. The absence of lowercase letter indicates no significant difference.

To investigate the expression profile of *PlewOBPs* after exposure to different tobacco volatiles, we carried out qRT-PCR experiments. The results showed that exposure to tobacco volatiles significantly altered the transcript abundance of several *PlewOBPs* (Figure 6). The transcript abundance of three *PlewOBPs* (*PlewOBP2*, *4*, and *12*) was considerably changed in male adults exposed to *S. litura*-infested tobacco. *PlewOBP2* was the only upregulated gene in this group compared to the blank control. Four *PlewOBPs* (*PlewOBP2*, *6*, *11*, and *13*) displayed significantly higher transcript abundance levels than the healthy tobacco exposure treatment. *PlewOBP4* had reduced transcript abundance in the IT group compared to the HT group. The transcript abundance of 5 *PlewOBPs*’ (*PlewOBP2*, *4*, *7*, *8*, and *12*) was significantly downregulated in female adults exposed to *S*. *litura*-infested tobacco compared to the blank control.

Compared to the HT group, *PlewOBP2*, *6*, *7*, and *8* exhibited significantly lower levels of transcript abundance, while *PlewOBP3* showed substantially higher levels of transcript abundance in the IT group. These numerously expressed *PlewOBPs* might be involved in regulating the behavioral activity of *P*. *lewisi* adults towards tobacco plants. The DREAM technique is the prevalent method for screening OBPs by examining substantial changes in mRNA expression levels upon exposure to odors and various biotic or abiotic factors (Chen et al., 2021). An study has shown that cucurbit chlorotic yellows virus (CCYV) infested *Bemisia tabaci* (Hemiptera: Aleyrodidae) exhibited increased orientation towards the host cucumber plant (He et al., 2023). Transcriptome analysis revealed 429 DEGs (407 upregulated and 22 downregulated) between CCYV-carrying and CCYV-free whitefly adults. Odorant-binding protein 5 (OBP5) was upregulated in CCYV-carrying whiteflies compared to CCYV-free ones and was proved to be involved in odor recognition and host localization. This transcriptional response is likely directly associated with the olfactory capabilities of OBPs, which may also aid insects in host location. In our study, differentially expressed OBPs between the IT and HT groups may be involved in the recognition progress of herbivore-induced plant volatiles released by *S*. *litura*-infested tobacco. However, this method is always associated with false positives or false negatives (Koerte et al., 2018). Additional tests, such as RNAi, are supposed to validate the results of this experiment in the future.

Transcription sequencing consistently reveals insights into the molecular mechanisms that regulate several physiological states in insects. For instance, Rinker et al. (2013) showed that the transcript abundance of *A. gambiae* odorant receptors (AgORs) and their excitatory odorant response profiles corresponded with the shift from host-seeking to oviposition behaviors in blood-fed female mosquitoes (Rinker et al., 2013). In contrast, AgOBPs exhibited a more complex pattern of variation, reflecting the multifaceted roles of OBPs, devoid of any apparent regularity. Besides olfactory-related genes, many genes from various signaling pathways also showed significant changes in transcript abundance, such as cytochrome P450 (Mappin et al., 2023). The molecular mechanisms behind the changes in transcript abundance induced by these genes remain unclear. These substances may possess additional physiological relevance for insects, or OBP may exhibit a diminished interaction with other signaling pathways. Nonetheless, our study demonstrates that the alterations in gene transcript abundance resulting from odor exposure are preserved in insects.

In conclusion, both male and female *P*. *lewisi* demonstrated changes in their transcription profiles when exposed to healthy or *S*. *litura*-infested tobacco plant odor. The mRNA levels in *P. lewisi* showed a sex-dependent modification after exposure to odor. The mRNA expression profiles differed significantly between male and female adults. Transcription sequencing identified the presence of 15 *PlewOBPs*, and 8 showed significant changes in transcript abundance when exposed to *S*. *litura*-infested tobacco, compared to the blank control or healthy tobacco. These genes provide novel targets for functional characterization, which may, in turn, lead to the development of tools and strategies for insect behavior regulation.

## Data Availability

The data presented in the study are deposited in the NCBI repository (https://www.ncbi.nlm.nih.gov/), accession number SRR31441214.
