# Supplementary material for: Identification and odor exposure regulation of odorant-binding proteins in Picromerus lewisi
Source: Front Physiol. 2024 Dec 4;15:1503440. doi: 10.3389/fphys.2024.1503440 (PMC11652525; doi:10.3389/fphys.2024.1503440)
Supplement: Supplementary file 2 [file Table1.docx]

**Supplemental Materials**

Table S1. Summary of the head transcriptome analysis of *Picromerus lewisi*

|  | **Transcript** | **Unigene** |
| --- | --- | --- |
| Total Length (bp) | 245546948 | 80419634 |
| Sequence Number | 156008 | 75039 |
| Max. Length (bp) | 22015 | 22015 |
| Mean Length (bp) | 1573.94 | 1071.7 |
| N50 (bp) | 2835 | 1728 |
| N50 Sequence Number | 25712 | 11373 |
| N90 (bp) | 603 | 422 |
| N90 Sequence Number | 97602 | 52355 |
| GC% | 32.82 | 31.96 |


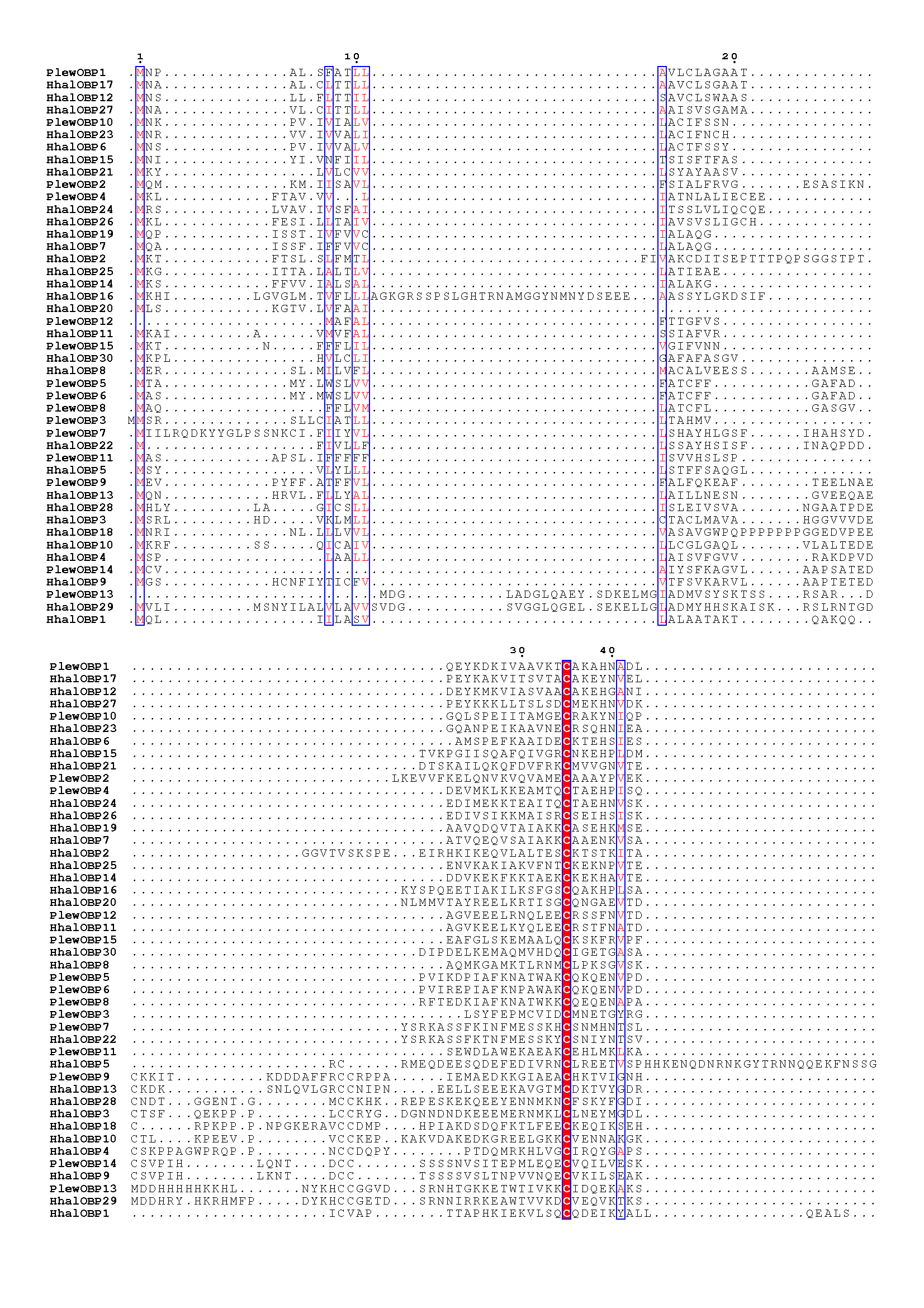


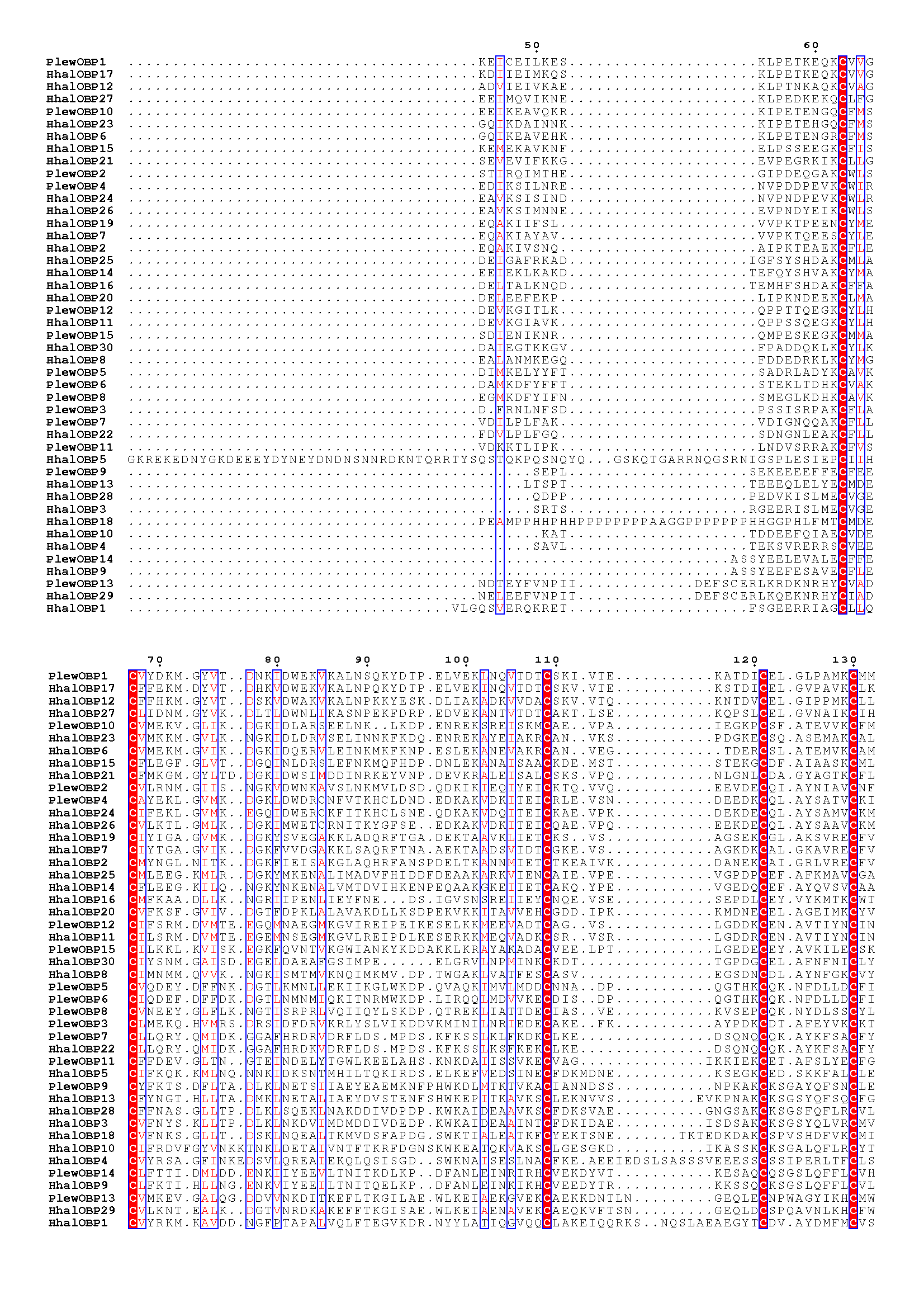


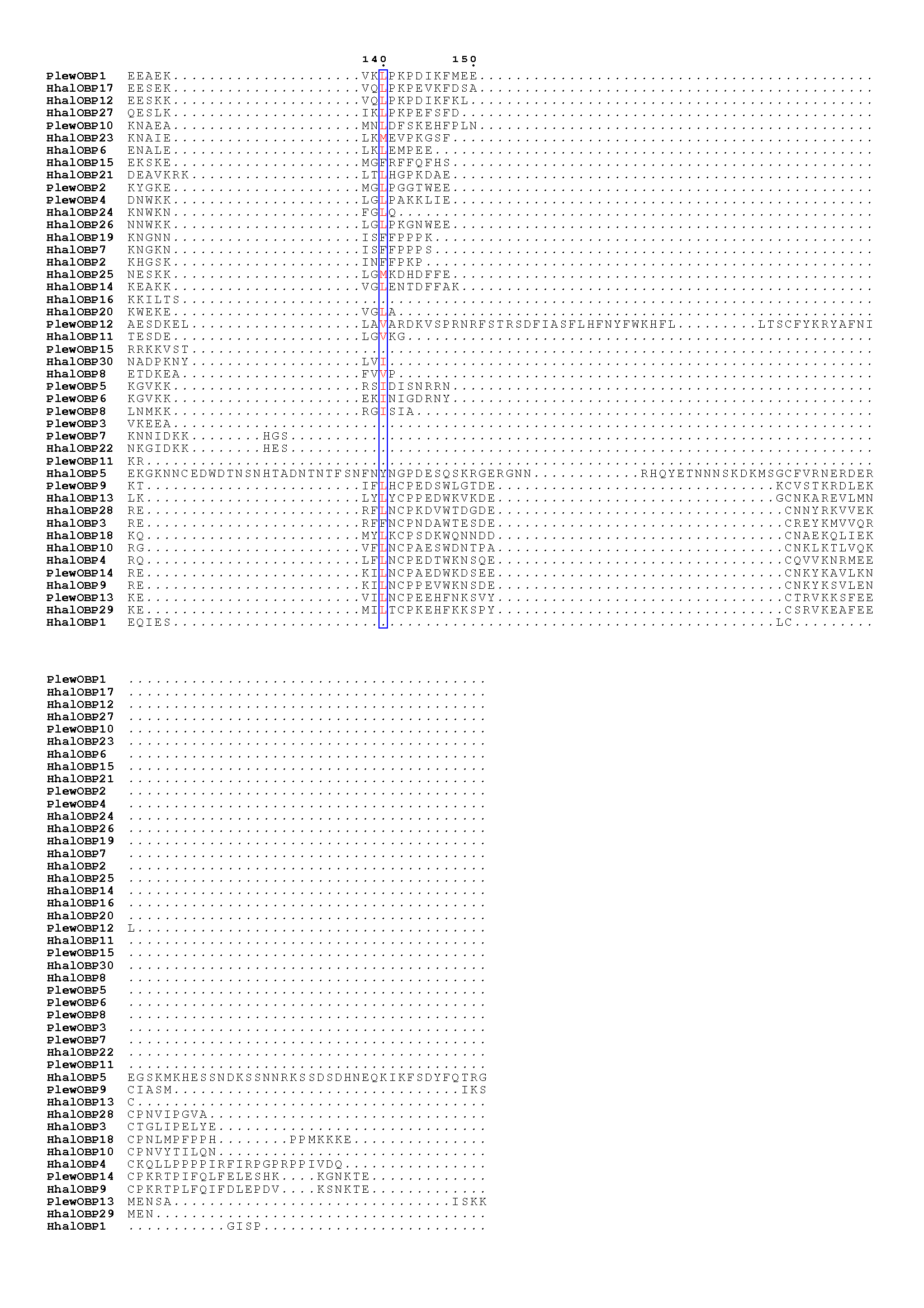


Figure S1. Multiple sequence alignment of OBPs from *Picromerus lewisi* and *Halyomorpha halys*


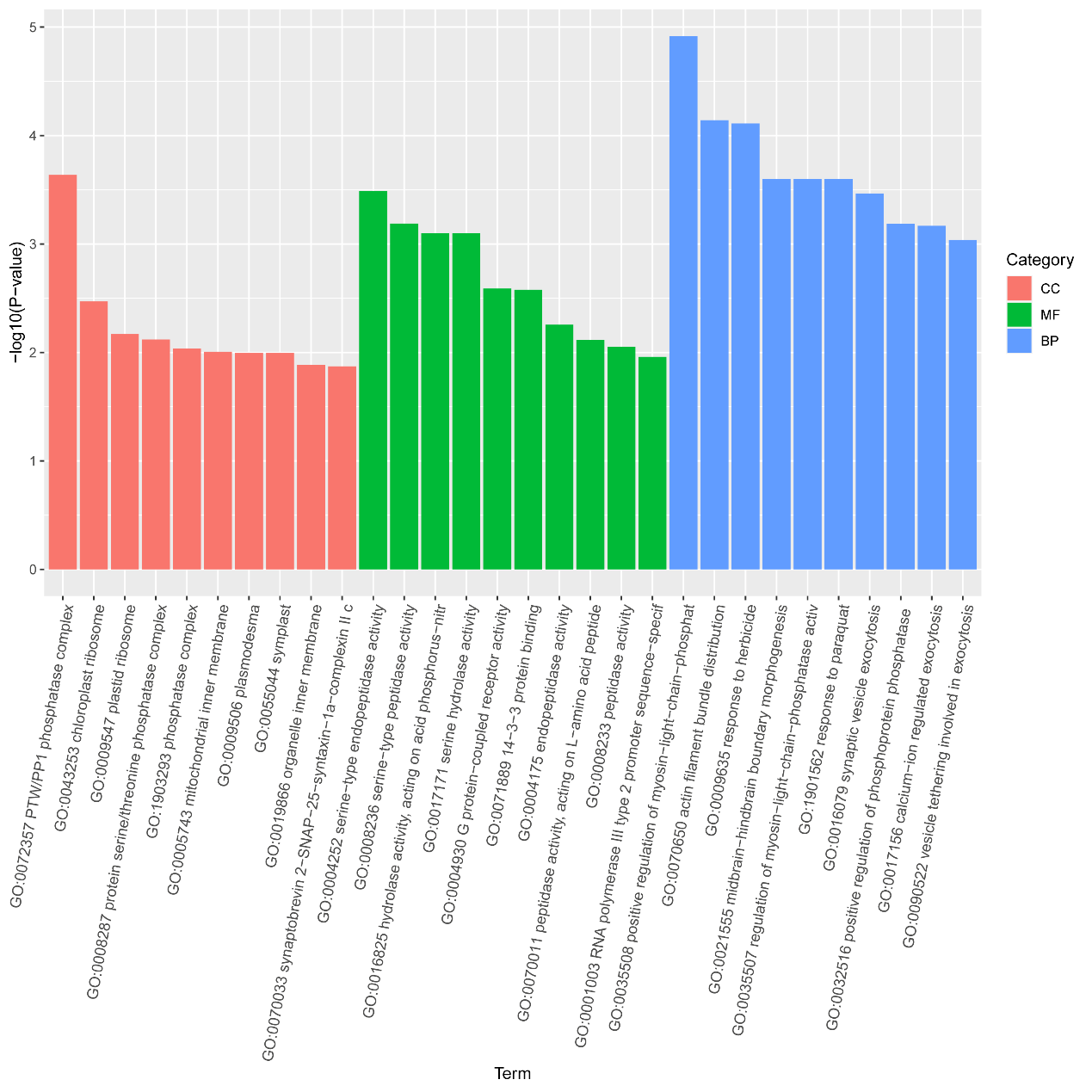
Figure S2. The GO enrichment patterns of female *Picromerus lewisi* after exposure to healthy tobacco.


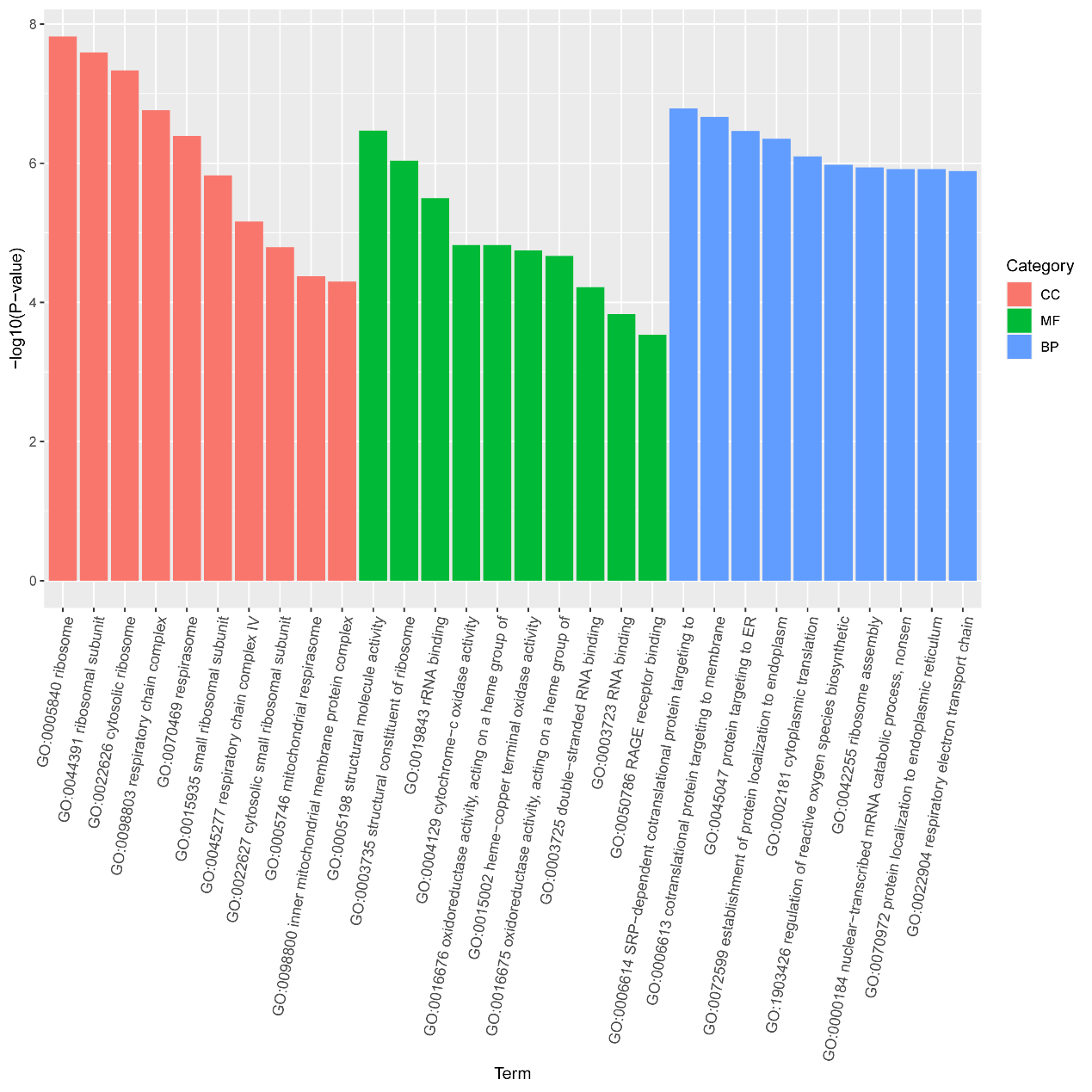
Figure S3. The GO enrichment patterns of male *Picromerus lewisi* after exposure to healthy tobacco.


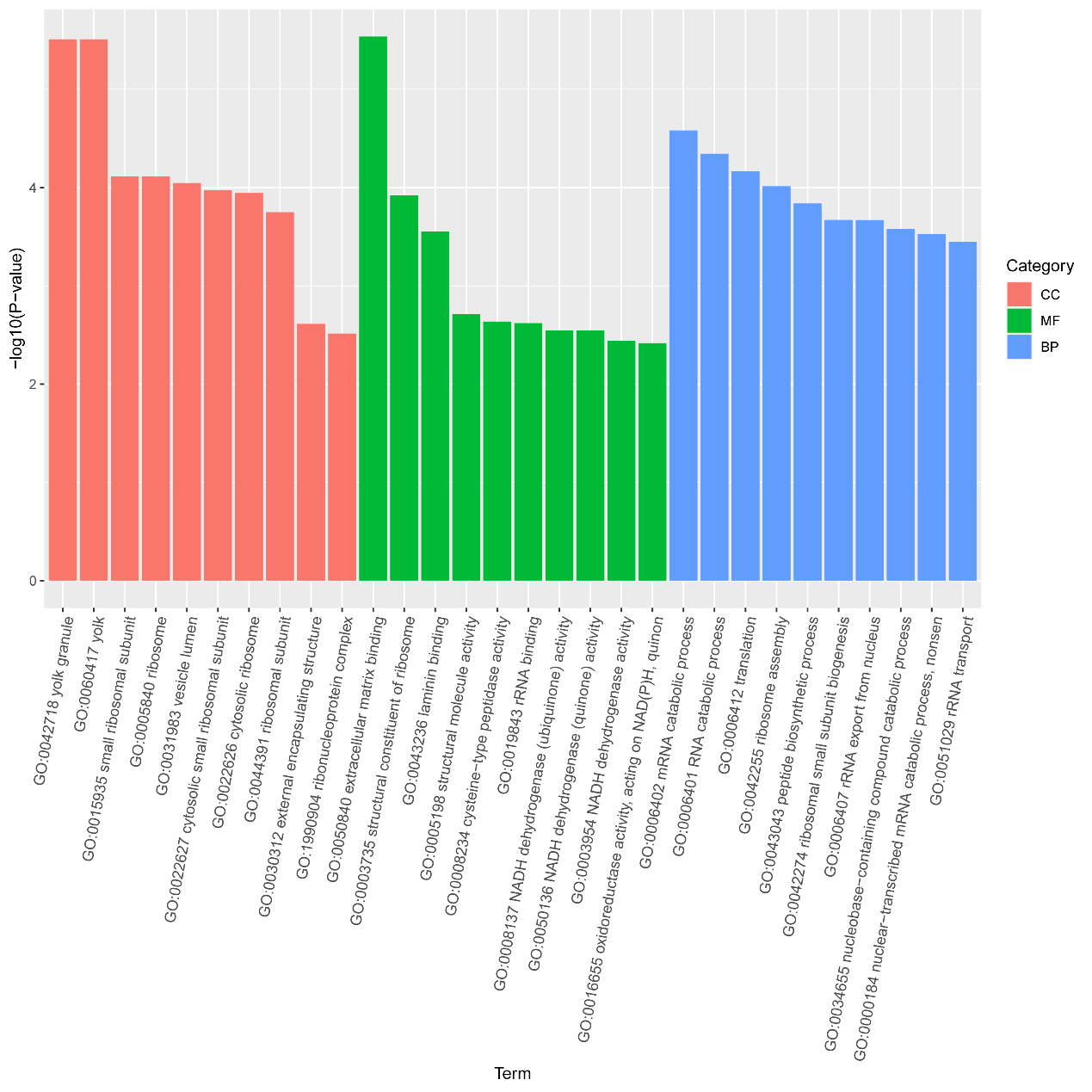
Figure S4. The GO enrichment patterns of female *Picromerus lewisi* after exposure to infested tobacco.


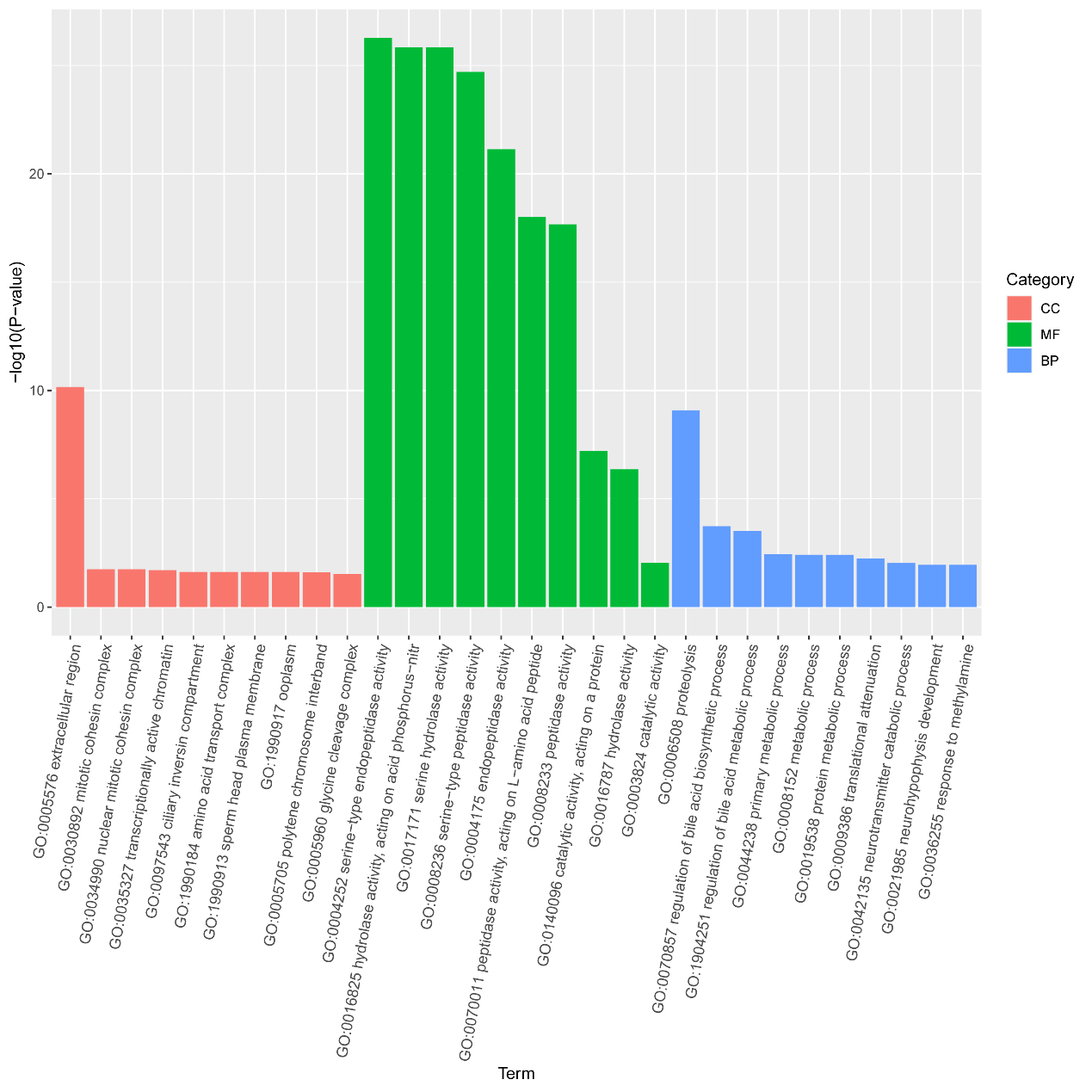
Figure S5. The GO enrichment patterns of male *Picromerus lewisi* after exposure to infested tobacco.
